# Potentially Clinically Relevant Drug–Drug Interactions in Oncology Patients Receiving Chronic Opioid Therapy: Prevalence and Associated Factors

**DOI:** 10.3390/life16081280

**Published:** 2026-08-02

**Authors:** Gorana Nedin Ranković, Dane Krtinić, Aleksandar Nikolić, Ana Cvetanović, Mirjana Todorović Mitić, Milica Mihajlović, Irena Conić, Nikola Milenković, Nemanja Dimić, Nada Pejčić, Iva Binić

**Affiliations:** 1 Department of Pharmacology with Toxicology, Faculty of Medicine, University of Niš, 18000 Niš, Serbia; dane.krtinic@medfak.ni.ac.rs; 2 Clinic of Oncology, University Clinical Center Niš, 18000 Niš, Serbia; ana.cvetanovic@medfak.ni.ac.rs (A.C.); mirjanatdrvc@gmail.com (M.T.M.); milica.radic@medfak.ni.ac.rs (M.M.); irena.conic@medfak.ni.ac.rs (I.C.); 3 Department of Surgery and Anesthesiology with Reanimatology, Faculty of Medicine, University of Niš, 18000 Niš, Serbia; dracanikolic@gmail.com; 4 Clinic for Anesthesia and Intensive Care, University Clinical Center Niš, 18000 Niš, Serbia; nada.pejcic@gmail.com; 5 Department of Oncology, Faculty of Medicine, University of Niš, 18000 Niš, Serbia; 6 Department of Infectious Diseases and Epidemiology, Faculty of Medicine, University of Niš, 18000 Niš, Serbia; milenkovic@dr.com; 7 Clinic for Infectious Diseases, University Clinical Center Niš, 18000 Niš, Serbia; 8 Clinic for Anesthesiology, Reanimatology and Intensive Care, University Clinical Hospital Centre “Dr Dragiša Mišović-Dedinje”, 11040 Belgrade, Serbia; nemanjadimic1989@gmail.com; 9 Department of Psychiatry, Faculty of Medicine, University of Niš, 18000 Niš, Serbia; ivabinic@gmail.com; 10 Clinic for Psychiatry, University Clinical Center Niš, 18000 Niš, Serbia

**Keywords:** drug–drug interactions, oncology, opioid therapy, polypharmacy, Lexicomp, Medscape, clinical pharmacology

## Abstract

Background: Patients with malignant diseases receiving chronic opioid therapy are particularly susceptible to drug–drug interactions because of extensive polypharmacy, multimodal anticancer treatment, supportive care, and comorbidities. This study aimed to determine the prevalence of potentially clinically relevant drug–drug interactions and to identify factors associated with their occurrence. Methods: This exploratory observational cross-sectional pilot study included 49 adult oncology patients receiving chronic opioid therapy. Complete medication regimens were screened using the Medscape Drug Interaction Checker and Lexicomp. Lexicomp category D and X interactions were analysed descriptively. Because category D interactions were nearly universal and category X interactions were rare, the presence of at least one Medscape “Serious—Use Alternative” interaction was used pragmatically as the binary outcome for regression modelling. Potential predictors were assessed using univariable binary logistic regression and a parsimonious adjusted model with covariates selected using a clinically informed approach. Because of quasi-complete separation, Firth’s penalized-likelihood logistic regression was used as the primary adjusted analysis. Results: Lexicomp category D interactions were identified in 48 of 49 patients (98.0%), whereas category X interactions were present in 2 patients (4.1%). Medscape serious interactions were detected in 33 patients (67.3%). In the Firth-adjusted model, female sex was associated with lower odds of a Medscape serious interaction (adjusted OR 0.12, 95% CI 0.02–0.52), while cardiovascular disease was associated with higher odds (adjusted OR 5.16, 95% CI 1.27–26.31). Stage IV disease showed a positive but statistically non-significant association, and total medication count was not independently associated with the outcome in sensitivity analysis. Because of the small pilot sample and the resulting wide confidence intervals, these findings should be interpreted as exploratory and hypothesis-generating. Conclusions: Oncology patients receiving chronic opioid therapy had a high burden of potentially clinically relevant drug–drug interactions, predominantly Lexicomp category D interactions, warranting consideration of therapy modification and individualized monitoring rather than absolute avoidance. Regular medication review, use of complementary interaction databases, and individualized clinical assessment may improve pharmacotherapy safety in this vulnerable population.

## 1. Introduction

Drug–drug interactions (DDIs) are a major challenge in contemporary pharmacotherapy, particularly among patients exposed to multiple medications. A DDI may be defined as a modification in the magnitude or duration of the effect of one drug caused by the concomitant or sequential administration of another drug, food, herbal product, or other exogenous substance [1,2]. Their clinical consequences range from minor changes without apparent clinical relevance to reduced therapeutic effectiveness, severe toxicity, hospitalization, prolonged treatment, and death [2,3]. The burden of DDIs is closely linked to polypharmacy, most commonly defined as the concurrent use of five or more medications [4]. Although polypharmacy is often clinically justified in patients with complex disease, the probability of potential interactions and adverse drug reactions rises substantially as the number of prescribed agents increases [4,5].

DDIs are generally classified as pharmacokinetic or pharmacodynamic. Pharmacokinetic interactions occur when one drug alters the absorption, distribution, metabolism, or elimination of another, thereby changing systemic exposure or drug concentration at the site of action. Pharmacodynamic interactions occur when two or more agents act on the same or related physiological systems, resulting in additive, synergistic, or antagonistic effects [6,7]. Both mechanisms may compromise treatment effectiveness or increase toxicity, especially when drugs with narrow therapeutic indices, overlapping adverse-effect profiles, or shared metabolic pathways are used together [8]. The clinical relevance of a database-generated interaction therefore depends not only on its formal severity category, but also on the dose, treatment duration, patient vulnerability, and the availability of preventive measures such as dose adjustment and laboratory or clinical monitoring.

Patients with cancer are particularly susceptible to DDIs because their treatment regimens are often complex and change over time. Antineoplastic therapy is frequently combined with analgesics, antiemetics, corticosteroids, anticoagulants, antimicrobials, psychotropic drugs, and medications for chronic comorbidities [9,10]. In addition, many patients use over-the-counter medicines, herbal products, or dietary supplements that may interact with anticancer and supportive care drugs [10]. Previous research has shown that potential DDIs are common in oncology, although reported prevalence varies considerably depending on the clinical setting, patient age, treatment type, number of drugs assessed, and the interaction database and severity definitions used [9,11,12]. This variability highlights the need to distinguish between all potential DDIs, interactions requiring closer monitoring or therapy modification, and combinations that should be avoided.

Polypharmacy is especially pronounced in patients with advanced cancer and cancer-related pain. In a large European cross-sectional study of 2282 patients with advanced cancer and pain, extensive medication use was common, reflecting the simultaneous need for disease-directed treatment, symptom control, and management of comorbidities [13]. In older patients with cancer receiving chemotherapy, polypharmacy and DDIs have likewise been reported at high rates, with greater medication burden and clinical complexity contributing to increased risk [14]. The number of possible drug pairs increases rapidly as medication count rises; consequently, patients receiving ten or more drugs may have dozens of potential pairwise combinations requiring assessment. Nevertheless, a high prevalence of software-detected interactions should not automatically be interpreted as an equally high prevalence of contraindicated prescribing, because many alerts represent manageable risks or clinically intentional treatment combinations.

Opioid analgesics remain central to the management of moderate-to-severe cancer pain, particularly in advanced disease [15,16]. Their use, however, creates additional opportunities for clinically relevant interactions. Pharmacodynamic interactions are especially important when opioids are combined with other central nervous system depressants, including benzodiazepines, gabapentinoids, sedative antidepressants, antipsychotics, or alcohol. Such combinations may increase sedation, cognitive impairment, falls, and respiratory depression. Pharmacokinetic interactions may alter opioid exposure and analgesic response through effects on phase I metabolism, phase II conjugation, or drug transporters such as P-glycoprotein [17,18,19]. The clinical impact may be particularly pronounced in frail patients, those with organ dysfunction, or those receiving several interacting drugs simultaneously.

Cytochrome P450 enzymes, particularly CYP3A4 and CYP2D6, play a central role in the metabolism of several commonly used opioids [17,20]. Fentanyl, oxycodone, and methadone may be affected by CYP3A4 inhibitors or inducers, which can respectively increase opioid exposure or reduce analgesic efficacy, whereas codeine and tramadol depend partly on CYP2D6-mediated formation of active metabolites. Genetic variability in CYP2D6 activity therefore contributes further interindividual variability in both efficacy and toxicity [16,17,18,21,22,23].

Several patient- and treatment-related factors may modify the risk of clinically important DDIs in oncology. These include older age, comorbidity burden, impaired hepatic or renal function, advanced disease, poor functional status, exposure to drugs with narrow therapeutic indices, and concomitant use of enzyme inhibitors or inducers [11,17,24]. The type of anticancer treatment and the intensity of supportive care may also influence interaction burden. Furthermore, centrally acting medications may have cumulative effects in patients with advanced cancer, and recent evidence suggests that central nervous system medication burden and interacting drug combinations may contribute to delirium and other unfavorable outcomes [25]. Identifying modifiable risk factors is therefore essential for improving the safety of cancer pain management and for supporting rational medication review.

Electronic interaction-checking systems are useful screening tools, but their output must be interpreted clinically: a category indicating that therapy modification should be considered is not equivalent to a contraindication. Studies evaluating DDIs should therefore report database-specific categories transparently rather than pooling distinct severity levels under a single label of ‘serious interactions.’

Despite the recognized burden of polypharmacy and DDIs in patients with cancer, evidence specifically focused on patients receiving chronic opioid therapy remains limited. This population combines several major risk determinants: advanced malignant disease, high medication burden, comorbidities, supportive care requirements, and exposure to drugs capable of producing additive central nervous system depression or clinically relevant metabolic interactions. The present study therefore aimed to determine the prevalence and characteristics of potentially clinically relevant DDIs in patients with malignant diseases receiving chronic opioid therapy and to identify sociodemographic, clinical, and treatment-related factors associated with their occurrence. Particular attention was paid to distinguishing interactions requiring therapy modification from combinations that should be avoided, and to evaluating serious interactions according to a separate interaction database.

## 2. Materials and Methods

### 2.1. Study Design and Participants

This exploratory observational cross-sectional pilot study included 49 adult patients with malignant diseases receiving chronic opioid therapy for cancer-related pain. The study was conducted at the Oncology Clinic of the University Clinical Centre Niš, Serbia, between March and June 2026. Eligible patients were 19–80 years old, had complete current medication records, and provided written informed consent. Chronic opioid therapy was defined as opioid use on a daily or near-daily basis for 90 days or longer; this criterion was applied clinically at the time of enrolment. Patients with severe cognitive or psychiatric impairment, an acute severe clinical condition, or incomplete medical documentation were excluded.

This cross-sectional study was reported in accordance with the Strengthening the Reporting of Observational Studies in Epidemiology (STROBE) statement [26].

### 2.2. Data Collection

Data were obtained from medical records and patient interviews. The complete medication history systematically included prescription medicines, over-the-counter medications, herbal products, and dietary supplements for all participants. All reported products were included in the drug–drug interaction screening when sufficient product information was available. Recorded variables included age, sex, place of residence, education, living arrangement, smoking status, coffee and alcohol consumption, primary malignancy, disease stage, comorbidities, Karnofsky Performance Status, total number of medications, opioid therapy, chemotherapy, radiotherapy, and other relevant anticancer and supportive treatments.

### 2.3. Assessment of Drug–Drug Interactions

The complete medication regimen of each patient was screened using the Medscape Drug Interaction Checker (https://reference.medscape.com/drug-interactionchecker) and Lexicomp (https://www.wolterskluwer.com/en/solutions/lexicomp). Interaction screening was performed between 11 and 16 July 2026. Both resources are continuously updated web-based databases without discrete version numbers; therefore, the classifications reported here reflect the content available during this access period. Medscape interactions classified as “Serious—Use Alternative” were recorded. In Lexicomp, category D interactions (“Consider Therapy Modification”) and category X interactions (“Avoid Combination”) were recorded and analysed separately.

Lexicomp and Medscape were selected as complementary, action-oriented screening tools because they provide explicit severity classifications and management recommendations and were accessible to the study team. Lexicomp uses risk-rating categories A, B, C, D, and X; category D indicates that therapy modification should be considered, whereas category X indicates that the combination should generally be avoided. Medscape grades interactions from minor or significant to “Serious—Use Alternative” and contraindicated. Micromedex and Drugs.com are valuable alternatives but were not available to the institution during data collection. Because databases differ in drug coverage, evidence thresholds, and classification, their categories were analysed separately and were not considered directly equivalent [27,28].

Automated interaction checkers may generate high alert volumes and do not fully account for dose, route, duration, laboratory findings, or overall clinical context; flagged combinations may therefore be intentional and clinically appropriate, particularly in oncology [29]. Database-generated alerts were interpreted as signals requiring clinical assessment rather than confirmation of a manifested DDI. The interaction mechanisms and management recommendations discussed in this study were derived from the databases and supporting pharmacological literature and were not measured in individual participants. No systematic data on clinical outcomes attributable to the identified interactions were collected.

### 2.4. Statistical Analysis

Categorical variables are presented as numbers and percentages, while continuous variables are reported as mean ± standard deviation or median with interquartile range, as appropriate. Associations between potential predictors and the presence of at least one Medscape serious interaction were examined using univariable binary logistic regression. The Medscape outcome was selected pragmatically after inspection of the interaction-category distributions because Lexicomp category D interactions were nearly universal and category X interactions were rare; it was not a pre-specified primary endpoint. Candidate variables for the adjusted model were selected using a clinically informed, parsimonious approach based on pharmacological plausibility and relevance to the study hypothesis. Univariable results were considered descriptively, but no fixed *p*-value threshold was used as a strict inclusion criterion. Female sex, cardiovascular disease, and stage IV disease were retained, and the number of covariates was deliberately restricted because of the limited number of outcome events (*n* = 33) and the exploratory nature of the study. Because the adjusted model showed a small-cell pattern consistent with quasi-complete separation (only 2 of 22 male participants did not have a Medscape serious interaction), Firth’s penalized-likelihood logistic regression was used as the primary adjusted analysis to reduce small-sample bias. The corresponding standard maximum-likelihood model was retained as a sensitivity comparison, and a further sensitivity analysis added total medication count as a covariate. Fourteen univariable associations were assessed without correction for multiple comparisons and were therefore interpreted as exploratory. Results are reported as odds ratios (ORs) with 95% confidence intervals (CIs). A two-sided *p* value < 0.05 was considered statistically significant. Statistical analyses were performed using Python version 3.13.5 (Python Software Foundation, Wilmington, DE, USA), with statsmodels (https://www.statsmodels.org/) used for standard logistic regression analyses. Firth’s penalized-likelihood logistic regression was performed in R version 4.6.1 (2026-06-24) using the logistf package (version 1.26.1) [30]. Confidence intervals and *p*-values for the Firth-penalized model are based on the profile penalized likelihood, as implemented by default in logistf.

### 2.5. Ethical Considerations

The study was approved by the competent Ethics Committee (approval No. 37288/10, dated 12 December 2024) and was conducted in accordance with the Declaration of Helsinki. All participants provided written informed consent.

Artificial intelligence use: Generative AI (Claude, Anthropic, Opus 4.8) was used to assist with drafting and language refinement of parts of the manuscript. It was not used for study design, data collection, statistical analysis, or interpretation of results. All AI-assisted text was verified and edited by the authors.

## 3. Results

### 3.1. Characteristics of the Study Population

The study included 49 adult oncology patients receiving chronic opioid therapy. The mean age was 66.7 ± 8.5 years, and 27 patients (55.1%) were women. The median number of concomitantly used medications was 12 (interquartile range [IQR] 10–14), while the mean Karnofsky performance score was 61.2 ± 17.5. Stage IV disease was present in 25 patients (51.0%). The main demographic and clinical characteristics are shown in Table 1.

### 3.2. Prevalence and Burden of Drug–Drug Interactions

Lexicomp category D interactions were identified in 48 patients (98.0%), with 97 D interactions in total and a median of 2 per patient (IQR 1–3). Category D denotes that therapy modification should be considered and was therefore analysed separately from category X. Lexicomp category X interactions, indicating that the combination should be avoided, were identified in 2 patients (4.1%), with 3 X interactions in total. Medscape classified at least one interaction as “Serious—Use Alternative” in 33 patients (67.3%), with 81 serious interactions overall. These findings are summarized in Table 2.

### 3.3. Most Frequent Lexicomp Interactions

The most frequent Lexicomp category D combinations are presented in Table 3. Carboplatin–paclitaxel and epirubicin–paclitaxel were intentionally co-administered as standard chemotherapy regimens; their category D classification reflects predictable overlapping toxicities and monitoring needs rather than inappropriate prescribing. Category D interactions do not necessarily require treatment discontinuation; rather, they indicate the need for dose adjustment, enhanced monitoring, or consideration of an alternative regimen. Only category X interactions represent combinations that Lexicomp recommends avoiding. Two distinct Lexicomp category X drug pairs were identified. The combination of fentanyl and enzalutamide occurred in two patients, while rivaroxaban and enzalutamide occurred in one patient, yielding three category X interactions in total. The identified Lexicomp category X interactions are summarized in Table 4.

### 3.4. Factors Associated with Medscape Serious Interactions

Because Lexicomp category D interactions were present in nearly all participants and category X interactions were rare, neither category provided sufficient outcome variability for a stable logistic regression model. Therefore, the presence of at least one Medscape “Serious—Use Alternative” interaction was used as the binary outcome. This was a pragmatic analytical choice made after inspecting the distribution of the interaction categories, rather than a pre-specified primary endpoint, and the modelling results should be read in that light. Univariable results are shown in Table 5. To reduce overfitting in this pilot sample, the primary adjusted model was restricted to female sex, cardiovascular disease, and stage IV disease and was estimated using Firth’s penalized logistic regression, as detailed in the Section 2.4.

Adjusted estimates are from Firth’s penalized-likelihood logistic regression (primary adjusted analysis). For comparison, the corresponding standard maximum-likelihood estimates were: female sex, OR 0.08 (95% CI 0.01–0.52, *p* = 0.008); cardiovascular disease, OR 6.47 (95% CI 1.28–32.64, *p* = 0.024); and stage IV disease, OR 4.62 (95% CI 0.92–23.26, *p* = 0.063). In a sensitivity analysis adding total medication count to the standard model, medication count was not independently associated with the outcome (OR 1.13, 95% CI 0.88–1.47, *p* = 0.343); cardiovascular disease and stage IV disease remained materially similar (OR 5.46, 95% CI 1.04–28.74, *p* = 0.045, and OR 4.54, 95% CI 0.90–22.95, *p* = 0.067, respectively). Univariable *p* values were not corrected for multiple comparisons.

In the Firth-adjusted model, female sex was associated with lower odds of a Medscape serious interaction (adjusted OR 0.12, 95% CI 0.02–0.52, *p* = 0.004), whereas cardiovascular disease was associated with higher odds (adjusted OR 5.16, 95% CI 1.27–26.31, *p* = 0.021). Stage IV disease showed a positive but statistically non-significant association (adjusted OR 3.80, 95% CI 0.92–19.38, *p* = 0.065). In a sensitivity analysis adding total medication count to the standard maximum-likelihood model, medication count was not independently associated with the outcome (adjusted OR 1.13, 95% CI 0.88–1.47, *p* = 0.343), while the associations with cardiovascular disease and stage IV disease remained materially similar. The confidence intervals remained wide; therefore, all adjusted estimates should be interpreted as exploratory and hypothesis-generating rather than confirmatory.

## 4. Discussion

This pilot study demonstrated a substantial burden of potential drug–drug interactions among patients with malignant diseases receiving chronic opioid therapy. Almost all participants had at least one Lexicomp category D interaction, whereas Lexicomp category X interactions were uncommon. In parallel, approximately two thirds of patients had at least one interaction classified by Medscape as “Serious—Use Alternative”. These findings indicate that clinically relevant interaction signals are frequent in this highly complex population, but they also underline the importance of distinguishing between different levels of clinical actionability. In particular, a Lexicomp D classification does not indicate that a combination is contraindicated; rather, it recommends consideration of therapy modification, dose adjustment, substitution, or intensified clinical and laboratory monitoring. By contrast, category X represents combinations that should generally be avoided.

The prevalence of Lexicomp category D interactions in the present study was very high, with 48 of 49 patients having at least one such interaction. Although this proportion initially appears striking, it is biologically and clinically plausible in the context of the study population. All participants had cancer, were receiving chronic opioid therapy, and were exposed to extensive polypharmacy, with many patients taking ten or more medicines. The number of theoretically possible drug pairs increases rapidly as the number of concomitant medicines rises; therefore, even a single additional drug can markedly increase the probability that an electronic interaction checker will identify at least one potential interaction. Previous oncology studies have consistently shown that polypharmacy is common and is one of the strongest determinants of potential DDIs [9,10,13,14]. In a European cross-sectional study of 2282 patients with advanced cancer and pain who required strong opioids, the mean number of medicines was 7.8, more than one quarter of patients used at least ten medicines, and exposure to pharmacodynamic and pharmacokinetic interactions was frequent [13].

Our findings are also consistent with studies showing that the prevalence of DDIs in oncology varies widely depending on the population, treatment setting, medicines included, and the database and severity threshold applied. Van Leeuwen et al. reported potential DDIs in a substantial proportion of patients receiving oral anticancer therapy, but only a smaller subset was considered major or required clinical intervention [12]. Similarly, prospective evaluation of cancer patients has shown that the number of computer-generated alerts may considerably exceed the number of interactions judged clinically relevant after expert review [31]. In older patients with cancer receiving chemotherapy, Oliveira et al. reported high rates of polypharmacy and drug interactions, including a considerable proportion of severe interactions [14]. Therefore, the 98% prevalence observed in the present study should not be interpreted as evidence that almost all patients were receiving unsafe or contraindicated regimens. Rather, it reflects the sensitivity of the Lexicomp D category to treatment combinations that warrant active clinical consideration in a population with an exceptionally high medication burden.

The low prevalence of Lexicomp category X interactions provides an important counterbalance to the high category D prevalence. Only two patients had at least one category X interaction, indicating that combinations explicitly recommended to be avoided were rare. This distinction is clinically meaningful and should remain explicit throughout interpretation of the results. The pattern suggests that most interaction signals represented potentially manageable risks rather than absolute prescribing errors. In oncology and palliative care, some interacting combinations may be intentionally prescribed because the expected therapeutic benefit outweighs the potential risk, provided that the patient is appropriately monitored. Standard antineoplastic regimens, combinations of opioids with adjuvant analgesics, and supportive medicines may generate electronic alerts because of overlapping toxicity, sedation, bleeding risk, QT prolongation, or effects on drug metabolism. Such alerts remain important, but their clinical meaning depends on dose, indication, duration, patient vulnerability, and the availability of monitoring or alternative treatments [11,17].

Medscape classified serious interactions in 33 of 49 patients. The difference between the Medscape and Lexicomp findings is not unexpected. Drug-interaction databases differ in their content, evidence thresholds, terminology, severity classification, and recommended management. Comparative studies have demonstrated substantial disagreement between commonly used interaction checkers, and no single database identifies every clinically relevant interaction with perfect sensitivity and specificity [27,32]. The use of two databases in the present study can therefore be considered a methodological strength because it provides complementary perspectives. However, direct equivalence between Lexicomp category D, Lexicomp category X, and Medscape “Serious—Use Alternative” should not be assumed. The categories should be reported separately, as was done in this study.

In the regression analysis, female sex was associated with lower odds of having a Medscape serious interaction, whereas cardiovascular disease was associated with higher odds. The latter association is clinically plausible because patients with cardiovascular comorbidity commonly receive antithrombotic agents, antihypertensives, antiarrhythmics, diuretics, and other medicines with relevant pharmacodynamic or pharmacokinetic interaction potential. The inverse association with female sex should be interpreted with particular caution. Only 2 of the 22 male participants did not have a Medscape serious interaction, leaving just two outcome-negative observations in the male stratum. Although the association remained statistically significant after Firth penalization, the estimate is unstable and should not be interpreted as evidence of a protective effect of female sex. Residual confounding by malignancy type, comorbidity patterns, or medication exposure is plausible. This finding is therefore hypothesis-generating only and requires confirmation in larger cohorts. Stage IV disease showed a positive but statistically non-significant association.

The clinical implications of these findings are particularly relevant for patients receiving opioids. Opioids may participate in pharmacodynamic interactions with benzodiazepines, gabapentinoids, sedative-hypnotics, antidepressants, antipsychotics, and other central nervous system depressants, potentially increasing sedation, confusion, falls, and respiratory depression. Pharmacokinetic interactions may occur through CYP3A4, CYP2D6, glucuronidation, or transporter-mediated mechanisms and may either increase opioid exposure and toxicity or reduce analgesic efficacy [16,17]. For example, the bromazepam–oxycodone combination should prompt reassessment of treatment necessity, use of the lowest effective doses, and enhanced clinical and respiratory monitoring. The fentanyl–enzalutamide combination may require avoidance, close monitoring for breakthrough pain, or selection of an opioid less dependent on CYP3A4 metabolism because enzalutamide may reduce fentanyl exposure and analgesic efficacy. In patients with advanced cancer, these risks are further modified by frailty, organ dysfunction, reduced functional status, dehydration, infection, and rapidly changing treatment regimens. Consequently, detection of a potential interaction should prompt individualized clinical assessment rather than automatic discontinuation of an otherwise beneficial medicine. These mechanisms represent general pharmacological interpretations derived from interaction resources and were not directly measured in this cohort.

Several limitations should be acknowledged. First, the cross-sectional design identifies potential interactions at a single time point and cannot establish causality. No systematic data on clinical outcomes attributable to the identified interactions were collected; therefore, the study cannot determine whether any alert resulted in a manifested drug–drug interaction, toxicity, hospitalization, treatment failure, or another patient-level outcome. Second, the small convenience sample from a single oncology centre limits statistical power and generalizability. The small-cell pattern and the limited ratio of events to predictors also produced wide confidence intervals, so the adjusted estimates should be interpreted as preliminary. Third, 14 univariable associations were examined without adjustment for multiple comparisons; the corresponding *p* values should therefore be interpreted as exploratory and hypothesis-generating rather than confirmatory. Fourth, electronic databases identify potential rather than necessarily manifested interactions and may overestimate clinically important risk when indication, dose, treatment duration, laboratory monitoring, and prescriber intent are not considered. Fifth, database coverage was incomplete for some medicines used in local clinical practice. At the time of assessment, chlorpheniramine and edoxaban could not be identified in the Lexicomp search interface used, while bromazepam, cilazapril, gliclazide, acenocoumarol, fenoterol, and zopiclone could not be identified in the Medscape checker. These medicines could therefore not be evaluated uniformly across both resources, introducing a possibility of under-detection or differential classification. This limitation supports the use of more than one database together with expert clinical review.

Despite these limitations, the study has several strengths. Some findings confirm established patterns: extensive polypharmacy generates a high volume of interaction alerts, and different databases may classify the same drug pairs differently. The principal contribution of this study is its opioid-specific, complete-regimen focus. Screening encompassed anticancer therapy, supportive care, treatment of comorbidities, over-the-counter medicines, herbal products, and dietary supplements. Patients receiving chronic opioid therapy for cancer pain are particularly vulnerable to additive central nervous system depression and CYP-mediated changes in opioid exposure, yet this group is seldom evaluated as a distinct population with the entire medication regimen considered. The separate reporting of Lexicomp D and X categories also provided a more clinically meaningful description of interaction burden and avoided conflating manageable alerts with combinations that should be avoided.

In summary, the present study found a very high prevalence of Lexicomp category D interactions but a low prevalence of category X interactions, while Medscape serious interactions affected approximately two thirds of participants. The findings indicate that the dominant problem in this population is not widespread use of absolutely contraindicated combinations, but a high cumulative burden of potentially modifiable or monitorable interaction risks arising from extensive polypharmacy, multimodal cancer treatment, comorbidities, and chronic opioid use. Regular medication reconciliation, periodic reassessment of treatment necessity, careful monitoring of central nervous system depression and other overlapping toxicities, and involvement of a clinical pharmacologist or pharmacist may help improve medication safety in this population.

## 5. Conclusions

Patients with malignant diseases receiving chronic opioid therapy showed a high burden of potentially clinically relevant drug–drug interactions, predominantly Lexicomp category D interactions requiring consideration of therapy modification or enhanced monitoring. In contrast, Lexicomp category X combinations were uncommon. The high interaction burden occurred in the context of extensive polypharmacy, while cardiovascular comorbidity was independently associated with higher odds of a Medscape serious interaction. Regular medication review, use of complementary interaction databases, and individualized clinical assessment are essential to improve pharmacotherapy safety in this vulnerable population.

## Figures and Tables

**Table 1:** Baseline demographic and clinical characteristics of the study population (*n* = 49).

Characteristic	Value
Age, years	66.7 ± 8.5
Female sex	27 (55.1)
Living alone	16 (32.7)
Higher education	19 (38.8)
Current smoker	15 (30.6)
Former smoker	21 (42.9)
Coffee consumption	30 (61.2)
Alcohol consumption	5 (10.2)
Diabetes mellitus	11 (22.4)
Cardiovascular disease	30 (61.2)
Psychiatric diagnosis	11 (22.4)
Number of medications	12 (10–14)
Chemotherapy	46 (93.9)
Radiotherapy	31 (63.3)
Karnofsky performance score	61.2 ± 17.5
Stage IV disease	25 (51.0)

Data are presented as mean ± standard deviation, median (IQR), or *n* (%), as appropriate.

**Table 2:** Prevalence and burden of potentially clinically relevant drug–drug interactions.

Database/Category	Clinical Interpretation	Patients with ≥1, *n* (%)	Total Interactions
Lexicomp D	Consider therapy modification	48 (98.0)	97
Lexicomp X	Avoid combination	2 (4.1)	3
Medscape Serious	Use alternative	33 (67.3)	81

**Table 3:** Ten most frequent Lexicomp category D drug pairs, frequencies, and principal mechanisms or clinical concerns (*n* = 49).

Rank	Drug Pair	Patients, *n*/N (%)	Principal Mechanism or Clinical Concern
1	carboplatin + paclitaxel	6/49 (12.2)	Standard chemotherapy regimen; overlapping myelosuppression and neurotoxicity
2	pregabalin + tapentadol	3/49 (6.1)	Additive CNS and respiratory depression
3	epirubicin + paclitaxel	3/49 (6.1)	Standard chemotherapy regimen; overlapping myelosuppression and neurotoxicity
4	bromazepam + oxycodone	2/49 (4.1)	Additive CNS and respiratory depression
5	oxycodone + tapentadol	2/49 (4.1)	Additive opioid and CNS-depressant effects
6	fentanyl + pregabalin	2/49 (4.1)	Additive CNS and respiratory depression
7	aceclofenac + methotrexate	2/49 (4.1)	Reduced methotrexate renal clearance; increased toxicity
8	aceclofenac + ginkgo	2/49 (4.1)	Potential additive bleeding risk
9	lorazepam + tramadol	2/49 (4.1)	Additive CNS and respiratory depression
10	ondansetron + roxitromycin	2/49 (4.1)	Additive QT-prolonging potential

**Table 4:** Lexicomp category X drug pairs identified in the study population, frequencies, and principal mechanisms or clinical concerns (*n* = 49).

Drug Pair	Patients, *n*/N (%)	Principal Mechanism or Clinical Concern
fentanyl + enzalutamide	2/49 (4.1)	CYP3A4 induction; reduced fentanyl exposure and analgesic efficacy
rivaroxaban + enzalutamide	1/49 (2.0)	CYP3A4/P-glycoprotein induction; reduced anticoagulant exposure

**Table 5:** Univariable and Firth-adjusted logistic regression for the presence of at least one Medscape serious interaction.

Variable	Univariable OR (95% CI)	*p* Value	Firth-Adjusted OR (95% CI)	*p* Value
Age, per year	0.98 (0.92–1.06)	0.643	—	—
Female sex	0.09 (0.02–0.48)	0.004	0.12 (0.02–0.52)	0.004
Living alone	0.48 (0.14–1.68)	0.253	—	—
Higher education	4.08 (0.98–17.03)	0.054	—	—
Current smoker	0.96 (0.26–3.48)	0.946	—	—
Former smoker	2.07 (0.59–7.29)	0.257	—	—
Coffee consumption	0.92 (0.27–3.16)	0.898	—	—
Alcohol consumption	2.07 (0.21–20.19)	0.532	—	—
Diabetes mellitus	2.62 (0.50–13.92)	0.257	—	—
Cardiovascular disease	4.44 (1.25–15.82)	0.021	5.16 (1.27–26.31)	0.021
Psychiatric diagnosis	0.81 (0.20–3.30)	0.766	—	—
Number of medications, per drug	1.20 (0.96–1.50)	0.106	—	—
Chemotherapy	1.03 (0.09–12.32)	0.979	—	—
Radiotherapy	2.30 (0.67–7.86)	0.184	—	—
Karnofsky score, per 10 points	0.75 (0.52–1.08)	0.12	—	—
Stage IV disease	3.38 (0.95–12.02)	0.059	3.80 (0.92–19.38)	0.065

## Data Availability

The data presented in this study are available on request from the corresponding author. The data are not publicly available due to privacy and ethical restrictions.
